# Supplementary material for: Superconductivity in High-Entropy Alloy System Containing Tb
Source: Materials (Basel). 2025 Jun 11;18(12):2747. doi: 10.3390/ma18122747 (PMC12194958; doi:10.3390/ma18122747)
Supplement: Supplementary file 1 [file materials-18-02747-s001.zip › materials-3644996-supplementary.pdf]

*materials*

MDPI

*Article*

# Superconductivity in High-Entropy Alloy System Containing Tb

**Piotr Sobota [1,2], Bartosz Rusin [1,2], Daniel Gnida [2], Adam Pikul [2] and Rafał Idczak [1,*]**

1 Institute of Experimental Physics, University of Wrocław, pl. M. Borna 9, 50-204 Wrocław, Poland; piotr.sobota2@uwr.edu.pl (P.S.); bartosz.rusin@uwr.edu.pl (B.R.)

2 Institute of Low Temperature and Structure Research, Polish Academy of Sciences, ul. Okólna 2, 50-422 Wrocław, Poland; d.gnida@intibs.pl (D.G.); a.pikul@intibs.pl (A.P.)

* Correspondence: rafal.idczak@uwr.edu.pl

**Abstract:** Superconducting alloy containing terbium (Tb) and a reference without the lanthanide were synthesized. X-ray diffraction, scanning electron microscopy, energy dispersive X-ray spectroscopy, specific heat and magnetic measurements were used to investigate their structural and physical properties. The comparison with the reference determined the effect of Tb on the alloy's critical parameters and phase stability connected to the high entropy alloys core effects.

**Keywords:** high-entropy alloys; superconductivity; terbium

check for updates

Received: 29 April 2025
Revised: 5 June 2025
Accepted: 9 June 2025
Published: 11 June 2025

**Citation:** Sobota, P.; Rusin, B.; Gnida, D.; Pikul, A.; Idczak, R.;. Superconductivity in High-Entropy Alloy System Containing Tb. *Materials* **2025**, *18*, 2747. https://doi.org/10.3390/ma18122747

**Copyright:** © 2025 by the authors. Licensee MDPI, Basel, Switzerland. This article is an open access article distributed under the terms and conditions of the Creative Commons Attribution (CC BY) license (https://creativecommons.org/licenses/by/4.0/).

These few paragraphs describe the research into the synthesis of Ln-doped bcc HEA superconductors and a reasoning why $(VNb)_{0.67}(TiTbHf)_{0.33}$ was selected to be synthesized and investigated Alloys with the following chemical compositions and stoichiometries inspired by the first superconducting alloy with high entropy were designed:

1. $(NbTa)_{0.67}(TiPrHf)_{0.33}$
2. $(NbTa)_{0.67}(TiNdHf)_{0.33}$
3. $(NbTa)_{0.67}(TiLaHf)_{0.33}$
4. $(NbTa)_{0.67}(TiCeHf)_{0.33}$
5. $(NbTa)_{0.67}(ZrHfCe)_{0.33}$
6. $(NbTa)_{0.604}(TiZrHfPr)_{0.33}$
7. $(NbTa)_{0.67}(CePrNd)_{0.33}$
8. $(NbTa)_{0.67}(TiHf)_{0.22}Zr_{0.10}Pr_{0.01}$
9. $(VNb)_{0.67}(TiNdHf)_{0.33}$
10. $(VNb)_{0.67}(TiErHf)_{0.33}$

In the synthesis attempts of alloys 1-8, tantalum was rolled out into a thin film in which the other metals were wrapped. In syntheses 9 and 10, rolled vanadium was used instead of tantalum foil. Before synthesis, the oxide film was removed from the lanthanides using sandpaper.

In the case of alloying samples containing tantalum, the metals did not melt together. Instead, the sample melted slightly, but no characteristic spheres were formed. The current intensity or melting time did not affect the result. Neither changing the lanthanide (syntheses 1-5), increasing the entropy (synthesis 6) or even increasing the lanthanide content (synthesis 7) allowed the metals to successfully melt together.

Several weeks after a series of syntheses 1-8, it was noticed that a precipitate corresponding in color to the oxides of the lanthanides in question appeared on the samples

Since oxidation occurs at the surface, it can be concluded that during melting, the entire volume of lanthanides migrates to the surface of the sample. This type of behavior sometimes occurs in syntheses of metal alloys with drastically different melting points.

Lanthanides, although they have a $T_{melting}$ lower than the other metals used, the range between their $T_{melting}$ and $T_{boiling}$ (Table S1) is wide enough that the other metals should melt. Only the difference between the $T_{melting}$ of tantalum and the $T_{boiling}$ of lanthanides is relatively small, and their heats of melting and evaporation are much smaller, so that strong evaporation could occur when lanthanide is concentrated on the sample surface. But even then, with prolonged heating, the tantalum should warm up enough to start melting. Nevertheless, the appearance of the samples before and after melting in the furnace is virtually the same.

**Table S1.** Selected physical properties of metals used in attempts to synthesize HEA alloys with lanthanides

| Element | $T_{melting}$ (K) | $T_{boiling}$ (K) | $C_{melting}$ (kJmol$^{-1}$) | $C_{evaporation}$ (kJmol$^{-1}$) |
|---|---|---|---|---|
| Ti | 1941 | 3560 | 18.7 | 425 |
| Zr | 2128 | 4682 | 21 | 580 |
| Hf | 2506 | 4876 | 25.5 | 630 |
| V | 2183 | 3680 | 22.8 | 453 |
| Nb | 2750 | 5017 | 26.8 | 690 |
| Ta | 3290 | 5731 | 36 | 735 |
| La | 1193 | 3737 | 6.2 | 400 |
| Ce | 1071 | 3633 | 5.5 | 350 |
| Pr | 1204 | 3563 | 6.9 | 330 |
| Nd | 1294 | 3373 | 7.1 | 285 |
| Tb | 1629 | 3503 | 10.8 | 295 |
| Er | 1770 | 3141 | 19.9 | 285 |

The decisive factor by which samples containing lanthanides and tantalum (including the sample from synthesis 8 containing only 1 at.% Pr) cannot be melted in an arc furnace appears to be the low thermal conductivity of liquid lanthanides. For La, Ce, Pr and Nd, it is 9, 8, 15 and 11 WK$^{-1}$m$^{-1}$, respectively. In comparison, the thermal conductivity of solid tantalum and niobium is 57 and 54 WK$^{-1}$m$^{-1}$ respectively. Thus, molten lanthanides can form a mantle on the surface of the tantalum foil, which slowly evaporating prevents the metal from heating up. To test this hypothesis, it was decided to perform melts, where instead of Ta foil, V foil was used (syntheses 9 and 10). As a result of arc heating, all metals melted, and the sample took the shape of a ball. This indicates the following mechanism that occurred in syntheses 1-8. (i) The lanthanide melts first and begins to migrate to the surface without combining with other metals. Before it reaches there, the tantalum melts slightly. (ii) Once it reaches the surface in sufficient quantity, it forms a kind of mantle that does not allow enough heat to reach the other elements, which are cooled all the time by the water running in the copper electrode. (iii) Slow evaporation of the lanthanide takes place on the surface. At this point, the arc heating was terminated due to the lack of visible effects. (iv) Lanthanide on the surface in contact with air begins to oxidize during the storage.
